# Application of Natural Clinoptilolite for Ammonium Removal from Sludge Water

**DOI:** 10.3390/molecules26010114

**Published:** 2020-12-29

**Authors:** Stephan Wasielewski, Eduard Rott, Ralf Minke, Heidrun Steinmetz

**Affiliations:** 1 Institute for Sanitary Engineering, Water Quality and Solid Waste Management (ISWA), University of Stuttgart, Bandtaele 2, 70569 Stuttgart, Germany; eduard.rott@iswa.uni-stuttgart.de (E.R.); ralf.minke@iswa.uni-stuttgart.de (R.M.); 2 Faculty of Civil Engineering, University of Kaiserslau-tern, Paul-Ehrlich-Str. 14, 67663 Kaiserslautern, Germany; heidrun.steinmetz@bauing.uni-kl.de

**Keywords:** ammonia, ammonium recovery, Freundlich, intraparticle diffusion, isoelectric state, Langmuir, pseudo-second-order, Temkin, zeolite, high-strength wastewater, sludge liquor

## Abstract

Sludge water (SW) arising from the dewatering of anaerobic digested sludge causes high back loads of ammonium, leading to high stress (inhibition of the activity of microorganisms by an oversupply of nitrogen compounds (substrate inhibition)) for wastewater treatment plants (WWTP). On the other hand, ammonium is a valuable resource to substitute ammonia from the energy intensive Haber-Bosch process for fertilizer production. Within this work, it was investigated to what extent and under which conditions Carpathian clinoptilolite powder (CCP 20) can be used to remove ammonium from SW and to recover it. Two different SW, originating from municipal WWTPs were investigated (SW1: *c*_0_ = 967 mg/L NH_4_-N, municipal wastewater; SW2: *c*_0_ = 718–927 mg/L NH_4_-N, large industrial wastewater share). The highest loading was achieved at 307 K with 16.1 mg/g (SW1) and 15.3 mg/g (SW2) at 295 K. Kinetic studies with different specific dosages (0.05 g_CLI_/mg_NH4-N_), temperatures (283–307 K) and pre-loaded CCP 20 (0–11.4 mg/g) were conducted. At a higher temperature a higher load was achieved. Already after 30 min contact time, regardless of the sludge water, a high load up to 7.15 mg/g at 307 K was reached, achieving equilibrium after 120 min. Pre-loaded sorbent could be further loaded with ammonium when it was recontacted with the SW.

## 1. Introduction

In view of the world’s population growing from 7.6 billion in 2017 to estimated 9.4–10.2 billion people by 2050, but also in consideration of rising living standards, correlating with increasing meat consumption, an increase of food requirements by 50% between 2012 and 2050 is to be expected [1]. This increased demand cannot be satisfied by the utilization of new farmland alone, since most of it is not developed, too remote from potential markets, susceptible to pest infestation or new cultivated land would compete with the conservation of important ecosystems. Furthermore, potential arable land is limited to a small number of countries. Rather, increasing productivity and efficiency in agricultural production must contribute to meet the increased demand [2], resulting in a greater need for nutrients, especially nitrogen fertilizers.

Nowadays, the nutrition of half the world’s population is ensured by the Haber-Bosch process, which enables the synthesis of ammonia (NH_3_) for fertilizer production [3]. However, the production of NH_3_ requires a high amount of energy (10 kWh/kg NH_3_) [4]. Dawson and Hilton [5] calculated that 1.1% of the world’s energy consumption can be attributed to the production of fertilizers; 90% of it due to the production of nitrogen fertilizers.

On the other hand, ammonium has severe negative environmental impacts. Because of its eutrophication potential, ammonium contributes to the growth of biomass in water bodies. Under alkaline conditions, which can occur during the day if intensive photosynthesis takes place, ammonium dissociates to ammonia, which has a toxic effect on aquatic fauna even in low concentrations.

In wastewater treatment rejected sludge water from the dewatering process of anaerobically treated sludge causes significant additional ammonium loads for the biological treatment step [6], resulting in additional need for energy and space. Additionally, external carbon sources might be necessary for a stable nitrogen elimination process. Instead of elimination and high-energy expenditure, the ammonium should be recovered to partially substitute the increasing worldwide demand for NH_3_.

Recovery methods such as air stripping, bioelectrochemical systems, membrane separation, and ion exchange have been thoroughly investigated. However, these methods require additional chemicals as well as energy, and ammonia losses due to volatilization can occur [7].

The zeolite clinoptilolite (CLI) is known to be a very good ion exchanger, as it consists of a three-dimensional tetrahedral structure formed of AlO_4_^−^ and SiO4, connected by a shared oxygen atom. The micropores formed by this structure are fine enough to allow entry and exchange of cations and water molecules [8]. This ability is based on the substitution of SiO_4_ by AlO_4_^−^, leading to a negative charge in the structure, which has to be compensated by exchangeable cations such as Na^+^, K^+^, Ca^2+^, and Mg^2+^ [9]. In a previous study with Carpathian clinoptilolite powder (CCP 20), 21.0 meq/100 g Na^+^, 49.3 meq/100 g K^+^, 65.6 meq/100 g Ca^2+^, and 3.3 meq/100 g Mg^2+^ were exchanged with 136.9 meq/100 g NH_4_^+^ [10].

As soon as the exchange capability for ammonium is exhausted, CLI is proposed to be utilized as a slow-release fertilizer in agriculture [11] or regenerated by the use of sodium chloride, sodium carbonate, sodium bicarbonate, or sodium hydroxide solutions [12,13,14,15].

In a study investigating the adsorption of ammonium from different highly concentrated wastewaters, it was shown that elimination from leachate of a sewage sludge landfill (*c*_0_ = 11.12–115.16 mg/L NH_4_-N) was 10–20% lower than from a matrix-free solution [16]. It has been demonstrated, that ammonium from swine manure (*c*_0_ = 0.43 M/L NH_4_^+^ ≈ 6150 mg/L NH_4_-N) can be removed by means of CLI, but the load is reduced from 10 mg/g (matrix-free ammonium solution) to 2 mg/g (manure) due to the cations contained [17]. Furthermore, dilution of leachate from a municipal landfill (*c*_0_ = 2292 mg/L NH_4_-N) does not improve ammonium adsorption [18]. In addition, organic compounds that are not removed by activated carbon interfere with the sorption of ammonium from leachate (*c*_0_ = 820 mg/L NH_4_^+^ = 637 mg/L NH_4_-N) at CLI; the load increases when leachate is pretreated with activated carbon [19]. The results of these studies show that the composition of the medium to which CLI is applied might be decisive for the adsorption effect. Since the adsorption of ammonium from sludge water, has not been sufficiently investigated, the adsorption process in this complex medium is not well understood and the technical implementation is uncertain.

The objective of this study was to develop a deeper understanding of the factors influencing the sorption of ammonium from sludge water on powdered natural clinoptilolite.

## 2. Materials and Methods

### 2.1. Zeolite Samples and Chemicals

Since preliminary studied Slovakian CLI CCP 20 (CCP = Carpathian clinoptilolite powder) showed favorable sorption properties [10], it was employed for this study. It was obtained from the supplier Labradorit GmbH (Berlin, Germany), in a ground and sieved form (particle size smaller than 20 µm). The CLI was dried at 378 K for 24 h before use. No other pretreatment was conducted. The CCP 20 mainly consisted of Si (35.5% (*wt*/*wt*)), Al (5.4%), K (2.0%), Ca (1.6%), Fe (1.0%), Na (0.4%), Mg (0.3%), Ti (0.1%), Ba (0.08%), and Pb (0.001%), whereas Cr, Ni, As, Rb, Cd, Cs, Ba, Hg, and Tl were below the limit of detection.

NH_4_Cl (p.a.), NaOH (p.a.) and HCl (32%, p.a.) were obtained from VWR International (Radnor, PA, USA).

### 2.2. Sludge Water and Matrix-Free Solution

Sludge water from two different WWTPs with high concentrations of ammonium nitrogen (see Table 1) were investigated. The sludge water originated from the dewatering process of anaerobically stabilized sludge by means of a chamber filter press. During normal operation, this sludge water is recycled to the main treatment process.

SW1 originated from a WWTP treating mainly municipal wastewater using the activated sludge process with upstream denitrification. Iron chloride sulphate is employed for phosphate precipitation. Primary sludge from mechanical treatment, secondary sludge from the biological stage and precipitated sludge from phosphorus elimination are admixed and anaerobically digested. Before dewatering, the digested sludge is pre-thickened and subsequently dewatered by means of a chamber filter press. For the investigations, the sludge water was taken from the outlet of the chamber filter press.

SW2 originated from a WWTP with a high industrial wastewater share. The wastewater is treated by trickling filters and a downstream denitrification. A mixture of aluminum and iron salts is employed as precipitant for phosphate elimination. Sludge from primary and secondary treatment as well as from phosphate elimination are pre-thickened and subsequently anaerobically digested. Afterwards, the digested sludge is dewatered with a chamber filter press, the outlet of which was sampled to gain SW2 for the investigations. Of SW2, several samples were examined, which were taken at different times.

Matrix-free ammonium chloride solution (*c*_0_ = 1000 mg/L NH_4_-N) was prepared by dissolving NH_4_Cl in distilled water.

Table 1 summarizes the physical properties and constituents of the examined sludge waters.

Both sludge waters showed only minor differences in composition except of COD (chemical oxygen demand) and PO_4_-P. The COD concentration in SW1 was about twice as high as in SW2, but the dissolved COD concentration (filtered by 0.45 µm nylon membrane) was similar. Presumably, the retention of COD-causing particles in the sludge dewatering of SW1 was less efficient than in SW2. In addition, the concentration of PO_4_-P in SW1 was considerably higher (factor 10).

In Table 2 the elementary composition of the examined sludge waters is listed.

The investigated sludge waters contained very low concentrations of heavy metals. They differed in their concentration of potassium and calcium, whereas the sodium and magnesium concentrations were almost identical.

Cations competing with ammonium ions for sorption sites such as K^+^ and Na^+^ were present in lower concentrations (K^+^: SW1: 234 mg/L = 6.0 mmol/L; SW2: 87.5–91.0 mg/L = 2.2–2.3 mmol/L; Na^+^: SW1: 126 mg/L = 5.5 mmol/L; SW2: 129–132 mg/L = 5.6–5.7 mmol/L) as ammonium (SW1: 967 mg/L NH_4_-N = 69 mmol/L; SW2: 718–913 mg/L NH_4_-N = 51.3–65.2 mmol/L). Accordingly, ammonium was present in multiple excess (SW1: [NH_4_^+^]/[K^+^] ≈ 12; [NH_4_^+^]/[Na^+^] ≈ 13; SW2: [NH_4_^+^]/[K^+^] ≈ 24–30; [NH_4_^+^]/[Na^+^] ≈ 10–12).

The ion ratios of ammonium to potassium and sodium were more favorable (high ammonium surplus) than in leachate from a sewage sludge landfill ([NH_4_^+^]/[Na^+^] ≈ 2.7; [NH_4_^+^]/[K^+^] ≈ 14.6 [16]) and leachate ([NH_4_^+^]/[K^+^] ≈ 2.2; [NH_4_^+^]/[Na^+^] ≈ 0.9 [19]), whereas a larger ammonium surplus was set in synthetic wastewater ([NH_4_^+^]/[K^+^] ≈ 17.3; [NH_4_^+^]/[Na^+^] ≈ 6.9 [20]).

### 2.3. Experimental Design

#### 2.3.1. Isoelectric State of CLI and pH-Dependent Adsorption

Both sludge waters were adjusted to pH values ranging from 2 to 12 by HCl or NaOH prior to the experiment. A fixed specific sorbent mass at a ratio of 0.1 g CLI per mg NH_4_-N was employed. Sorbent (20 g) and solution (200 mL) were stirred for 20 h on a magnetic stirrer (400 rpm) at room temperature (295 K) in closed bottles, subsequently membrane-filtered (0.45 µm pore size), and then the pH value as well as the ammonium concentration were determined. The initial pH values of the solutions were compared with those of the filtrates. The isoelectric state is the point at which both pH values are identical.

Sodium ions, which are added to the sample by NaOH to adjust the pH, compete with ammonium for the sorption sites in the CLI. In order to show the influence of the sodium ions without additionally changing the pH value, equimolar NaCl was added. All experiments were conducted as triplicates. The titration curves of NH_4_Cl solution, SW1 and SW2 are also depicted in Appendix A.

#### 2.3.2. Isothermal Adsorption

Since sludge water originates from the digestion tower, operated moistly at mesophilic conditions, the influence of temperature on CLI loading was investigated. For this purpose, temperatures of 307 K (34 °C, mesophilic conditions in the digestion tower, considering small heat losses), 295 K (22 °C, room temperature) and 283 K (10 °C) were tested.

Since the ammonium concentration in the sludge water could not be changed, the amount of sorbent mass *m* (g) was varied instead. Thus, different quantities ranging from 2 g to 48 g sorbent were mixed with 200 mL sludge water *V_p_* (mL) and stirred at a constant temperature (283 K, 295 K, and 307 K) on a magnetic stirrer at 400 rpm. After 20 h, the residual ammonium concentration *c_eq_* (mg/L) as well as the pH in the filtrate were determined. Since the pH barely varied between the different dosages and a competing adsorption by Na^+^ or H_3_O^+^ cations as well as a dilution due to the pH adjustment was to be avoided, a pH correction was not conducted. One experimental approach without sorbent for each examined pH was used to determine unwanted ammonium elimination, e.g., by stripping or adsorption onto parts of the glass apparatus. All experiments were conducted as triplicates. The ammonium concentration in the filtrate of that approach is expressed as *c_B_* (mg/L). The loading *q_eq_* (mg/g) of the sorbent mass was determined by Equation (1).

(1) $$q_{eq}=\frac{\left(c_{0}-{(c}_{0}-c_{B})\;-\;c_{eq})\times (\frac{V_{P}}{1000}\right)}{m}$$

#### 2.3.3. Adsorption Kinetics

The influence of temperature (283 K–307 K; constant specific sorbent ratio of 0.1 g_CLI_/mg_NH4-N_; non pre-loaded CLI), the influence of the specific sorbent ratio (0.05–0.2 g CLI per mg NH_4_-N; constant temperature 295 K; non pre-loaded CLI), as well as the influence of pre-load (0–11.4 mg_NH4-N_/g_CLI_; constant sorbent ratio 0.1 g_CLI_/mg_NH4-N_; constant temperature 307 K) were investigated in kinetic experiments. Furthermore, a matrix-free ammonium chloride solution (*c*_0_ = 1000 mg/L NH_4_-N) was examined as well. The sorption properties of CCP 20, including isoelectric state, pH-dependent elimination, isothermal adsorption, and thermodynamic properties were already investigated with similar matrix-free solution by Wasielewski et al. [10].

CCP 20 was mixed with the sample (1.5 L) on a magnetic stirrer. All experiments were conducted as triplicates. At periodic intervals, an aliquot (10 mL) was taken and immediately membrane-filtered (nylon membrane, 0.45 µm pore size) to prevent further contact between sorbent (CLI) and sample (NH_4_Cl solution or sludge water). Subsequently, the ammonium concentration was measured in the filtrate and the time-dependent loading of the sorbent *q*(*t*) (mg/g) was calculated. Since it is known from published studies that the adsorption kinetics strongly depend on the stirring speed [21,22,23], a high rotation frequency of 800 rpm was chosen to determine the maximum possible adsorption kinetic values. Due to sampling during the test, the total volume was continuously reduced. However, it can be assumed that during the sampling no change in the ratio of the sorbent mass to the volume of the solution occurred due to the homogeneously mixed conditions.

### 2.4. Adsorption Models

#### 2.4.1. Freundlich Model

The nonideal, reversible adsorption of a heterogenous surface is described by the empirical Freundlich model [24]. It is not possible to calculate a complete loading, as the sorbent sites can be occupied in several layers. The loading of the sorbent *q_eq,F_* (mg/g) can be calculated by exponentiation of the corresponding equilibrium concentration *c_eq_* (mg/L) with the factor *1/n* (–), as described by Equation (2).

(2) $$q_{eq,F\;}{\;=\;K}_{F}\;c_{eq}^{\frac{1}{n}}$$

Calculation methods for determining the constants *K_F_* and *1/n* with the help of nonlinear regression or linearization are given, e.g., by Ho et al. [25]. In this study, the linearization was done by plotting log *q_eq_* versus log *c_eq_*. The gradient of the graph corresponds to *n*, while the tenth power of the intercept represents *K_F_*.

#### 2.4.2. Langmuir Model

A monomolecular layer of adsorbate on the available sorption sites is assumed according to the adsorption model of Langmuir [26]. Thus, the properties of the sorbent sites are identical and equivalent, so that a determination of the maximum adsorption capacity is possible. The loading of sorbent is calculated according to Equation (3), where *K_L_* (L/mg) is the Langmuir constant and *q_max_* (mg/g) the maximum capacity.

(3) $$q_{eq,L}\;=\;\frac{q_{max}K_{L}c_{eq}}{{1+K}_{L}c_{eq}}$$

The constants can either be deduced from linear or nonlinear regression based on measurement results. By plotting *c_eq_/q_eq_* vs. *c_eq_*, *1/q_eq_* vs. *1/c_eq_*, *q_eq_* vs. *q_eq_/c_eq_*, or *q_eq_/c_eq_* vs. *q_eq_*, a linear relationship for Equation (3) can be deduced [27]. Table 3 lists the four possible linear forms for determining Langmuir constants. In this study, only the type of isotherm with the highest coefficient of determination *r²* is listed. The coefficient of determination *r²* of the nonlinear form of the Langmuir isotherm and the experimentally determined loads *q_eq_* and the arithmetical average loads $\bar{q_{eq}}$ were calculated according to Equation (4).

(4) $$r^{2}\;=\;\frac{\sum^{\;}{\left(q_{eq,L}\;-\bar{q_{eq}}\right)}^{2}}{\sum^{\;}{\left(q_{eq,L}\;-\bar{{\;q}_{eq}}\right)}^{2}+\sum^{\;}{\left(q_{eq,L\;}-{\;q}_{eq}\right)}^{2}}$$

#### 2.4.3. Temkin Model

The isothermal loading of sorbents according to Temkin ([29] in [30]) is extended by the temperature parameter. Accordingly, the adsorption enthalpy is linearly proportional to the loading on the sorbent [31]. The form of the isotherm used in this work is taken from Ho et al. [25] (Equation (5)), where *R* is the universal gas constant (8.314459 J/(mol K)), *T* the temperature (K), *b_T_* (1/mol), and *A_T_* (L/mg) the Temkin isothermal constants.

(5) $$q_{eq,T}\;=\;\frac{RT}{b_{T}}ln\left(A_{T}c_{eq}\right)$$

The linearized form of the Temkin isotherm is shown in Equation (6).

(6) $$q_{eq,T}\;=\;\frac{RT}{b_{T}}ln\left(A_{T}\right)\;+\;\frac{RT}{b_{T}}ln\left(c_{eq}\right)$$

In a plot of ln *c_eq_* vs. *q_eq_*, the term *RT/b_T_* is represented by the slope, whereas the intersection with the ordinate represents the term *RT ln(A_T_)/b_T_*. Subsequently, *b_T_* and *A_T_* can be deduced.

#### 2.4.4. Thermodynamic Calculations

Energy adsorption or release, i.e., temperature increase or decrease, can be observed during the adsorption process. The standard free energy *∆G*^0^ (kJ/mol) can be calculated according to the following Equation (7)

(7) $${\Delta G}^{0}\;=\;-RTln\left(K_{d}\right)$$

where *K_d_* is the thermodynamic equilibrium constant, here the Freundlich constant (L/g). According to Milonjic [32], it should be noted that *K_d_* must be dimensionless. Therefore, the use of the temperature-dependent equilibrium constant *K_F_* must be corrected by a factor of 1000 g/L (density of water) into its dimensionless form. The relationship of the other thermodynamic parameters such as change in enthalpy *∆H*^0^ (kJ/mol) and change in standard entropy *∆S*^0^ (J/(mol K)) can be derived by means of the Gibbs–Helmholtz Equation (8).

(8) $${\Delta G}^{0}{\;=\;\Delta H}^{0}-{T\Delta S}^{0}$$

From the plot of the logarithmic equilibrium constant *K_d_* against the reciprocal value of the temperature *1/T* (Van’t–Hoff diagram), a linear correlation can be derived. Here, the gradient corresponds to the quotient of the negative change in the free standard enthalpy *∆H*^0^ and the universal gas constant *R*. Furthermore, the quotient of the change of the free molar standard entropy *∆S*^0^ and the universal gas constant can be derived from the axis section.

Endothermic adsorption is described by a positive value of *∆H*^0^, meaning energy is absorbed by the adsorption process. A negative value indicates exothermic adsorption, meaning energy is being released. A spontaneous (exergonic) adsorption is expressed by negative *∆G*^0^, while negative *∆S*^0^ indicates a random adsorption behavior.

### 2.5. Kinetic Models

#### 2.5.1. Intraparticle Diffusion

A mathematical description of the diffusion process is provided by the intraparticle diffusion model (ID). It presumes a correlation between the loading rate *k_ID_* (mg/(min^0.5^ g)) and the square root of the contact time *t* (min) ([33] in [34]). However, McKay et al. [35] extended this model by the constant *C* (mg/g), which is proportional to the thickness of the boundary layer as well as the initial adsorption by it. The time-dependent loading of the sorbent *q*(*t*)*_ID_* (mg/g) can be calculated by Equation (9).

(9) $$q{\left(t\right)}_{ID}{\;=\;k}_{ID}t^{0.5}\;+\;C$$

To determine the loading rate *k_ID_*, *q*(*t*) versus *t*^0.5^ is plotted. The slope of the resulting graph corresponds to *k_ID_* while the intersection with the ordinate corresponds to *C*. Sole intraparticle diffusion occurs when the graph intersects the origin (*C* = 0). If a multistage diffusion process is present, two or more partial lines passing into each other can be approximated to the existing empirical measuring points of *q*(*t*).

#### 2.5.2. Pseudo-Second-Order

The time-dependent loading of the sorbent can be described by the pseudo-second-order (PSO) model according to Ho and McKay [36]. However, it is not possible to deduce the prevailing adsorption kinetic processes when using this model. It offers a macroscopic view of the adsorption process, based on the assumption that the adsorption rate is dependent on the loading of the ion exchange material at a certain point in time and its equilibrium state. The differential form of the PSO, i.e., as the differential of the load *q*(*t*) (mg/g) at any time *t,* is given in Equation (10)

(10) $$\frac{dq_{t,PSO}}{dt}{\;=\;k}_{2}{\left(q_{e}\;-{\;q}_{t}\right)}^{2}$$

where *k*_2_ is the pseudo-second-order rate (mg/(g min)) and *q_e_* (mg/g) the load at equilibrium. From the integration of Equation (10) with the boundary conditions *q*(*t*) = 0 at *t* = 0 and *q*(*t*) = *q*(*t*) at *t* = *t*, four different linear forms of the PSO model can be obtained (Table 4).

In this study, only the type with the highest coefficient of determination *r*^2^ (Equation (4)) is listed. All calculations in this study were conducted using Microsoft Excel 2019.

### 2.6. Analytical Methods

Ammonium was measured according to German standard DIN 38406-5 [38]. At a pH of about 12.6, ammonium cations and ammonia contained in the sample react with hypochlorite ions and salicylate ions in the presence of sodium pentacyanonitrosylferrate (2-)(nitroprusside sodium) as a catalyst to form a blue dye. The required hypochlorite ions are formed in the alkaline medium by hydrolysis of the dichloroisocyanuric acid ions. The spectral absorbance of the blue dye at 655 nm wavelength is linearly proportional to the ammonium concentration.

For determination of pH, probes (SenTix 950 + Multi 3430, WTW, Weilheim, Germany) were used.

F^-^, Cl^-^, NO_2_-N, NO_3_-N, Br^-^, SO_4_^2-^, and PO_4_-P were analyzed according to ISO 10304-1 [39] using the Dionec ICS-110 ion chromatograph (Thermo Fischer Scientific, Waltham, MA USA). Before the determination, the sample was filtered through a C18 cartridge (Strata C18-E (55 µm, 70 Å), Phenomenex, Torrance, USA) and diluted if necessary.

To determine the elementary composition of the sludge waters, 44 mL of sample were admixed with 2 mL HCl (32%), 3 mL HNO_3_ (65%), 1 mL H_2_O_2_ (30%) and digested by a microwave (Start, MLS GmbH, Leutkirch, Germany) with a selected program run of 10 min at 443 K and a subsequent cooling phase of 20 min.

To determine the chemical elements of the zeolite, 0.3 to 0.5 g of the CLI were weighed and mixed with 6 mL HNO_3_ (65%), 4 mL HF (48%), and 2 mL HCl (32%). The mixture was digested by microwave with a selected program run of 10 min at 383 K, then 5 min at 413 K, and finally 9 min at 463 K. Together with the cooling phase, the digestion lasted 64 min.

Heavy metals were analyzed by inductively coupled plasma mass spectrometry (Nexion 2000, Perkin Elmer, Waltham, MA, USA).

## 3. Results and Discussion

### 3.1. Isoelectric State of CLI and pH-Dependent Adsorption

**Figure 1:**
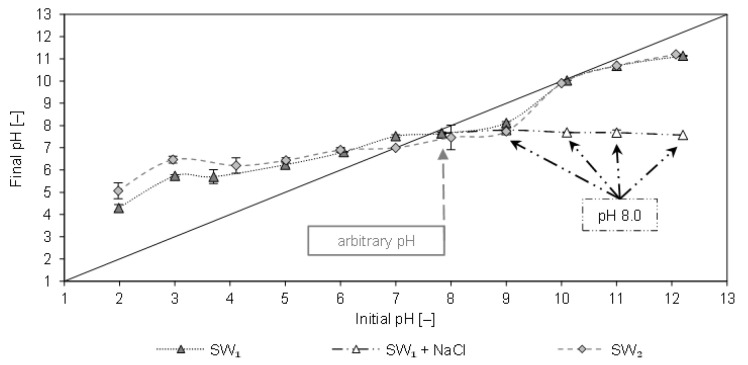
Final pH of the filtrates after 20 h contact with CCP 20 (SW1: *c*_0_ = 967 mg/L NH_4_-N; SW2: *c*_0_ = 927 mg/L NH_4_-N; *T* = 295 K; sorbent ratio 0.1 g_CLI_/mg_NH4-N_) as a function of the initial pH (adjusted pH of the solution before contact with CLI).

In Figure 1 the final pH of SW1 and SW2 filtrates are plotted as a function of the initial pH after contact with CCP 20. The arbitrary pH value of SW1 was 7.9 and that of SW2 was 8.0. In the alkaline range, no considerable change in the pH was observed. This can be attributed to the decrease in ammonium uptake, as uncharged NH_3_ is formed at pH >8, which is not adsorbed by the CCP 20. As a result, no cations are eluted that could lead to a change in the pH.

The pH increased in the acidic range (2–6), which can be attributed to the removal of NH_4_^+^ and the elution of cations (e.g., Na^+^, K^+^, Ca^2+^, and Mg^2+^). The isoelectric state (pH_ISO_) of CCP 20 with both sludge waters occurred at pH values of 8 and 10. The same values of pH_ISO_ were also determined after contact of CCP 20 with matrix-free NH_4_Cl solution [10]. Hence, an influence of the sludge water matrix on the pH_ISO_ is not detectable. Furthermore, Figure 1 shows the pH after spiking SW1 with equimolar NaCl instead of NaOH; the latter was added to the sample to adjust the pH. When spiked with NaCl (23–168 mmol/L), the pH remained at approx. 8.

**Figure 2:**
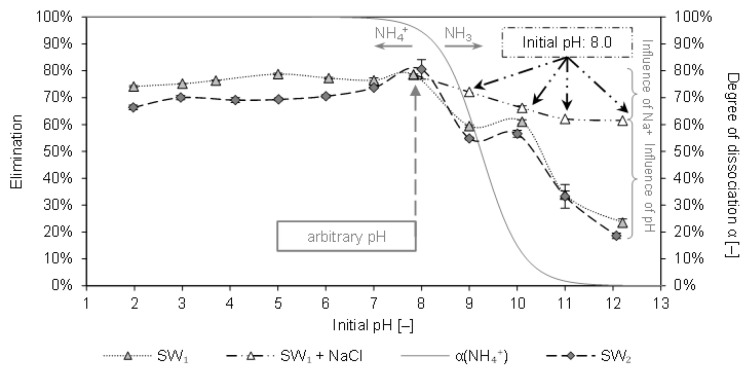
Elimination of ammonium from sludge water by CCP 20 (SW1: *c*_0_ = 967 mg/L NH_4_-N; SW2: *c*_0_ = 927 mg/L NH_4_-N; *T* = 295 K; sorbent ration 0.1 g_CLI_/mg_NH4-N_) after 20 h contact time as a function of different initial pH of the sludge water (adjusted pH before contact with CCP 20); in addition, the influence of an equimolar amount of Na^+^ (from NaCl instead of NaOH to raise the pH) on the elimination is shown; degree of dissociation of ammonium in grey.

Figure 2 depicts the elimination of ammonium from SW1 and SW2 by CCP 20 as a function of the initial pH. Furthermore, the degree of dissociation α of the NH_4_^+^/NH_3_ system is plotted over the initial pH.

In the pH-range from 2 to 8 a consistent elimination between 66% and 81% was determined. A decrease in the elimination could be observed with pH values above 8. For SW1, the elimination decreased from 79% at pH 8 to 58% at pH 9 (59% at pH 10) and dropped to 24% at pH 12.2. At the same pH, the elimination from SW2 decreased from 81% to 55% (57% at pH 10) and finally to 19% (at pH 12). The batches with NaCl instead of NaOH indicate that the influence of sodium ions competing for sorption sites on the elimination is inferior to the influence of the pH. Although the elimination already declined from 79% to 62% due to the addition of Na^+^ (from NaCl), the equimolar amount of NaOH, which increased the pH to 12.2, led to an even lower elimination of only 24%.

When comparing these results with results from similar experiments with matrix-free solution (pH 9: 73% elimination; pH 12.2: 20% elimination [10]), only a slightly negative influence of the SW matrix becomes apparent. Table 2 shows that both SW1 and SW2 had constituents (e. g., K^+^, Na^+^) that compete with ammonium for adsorption sites

Comparable studies with leachate have reported an elimination of 68% at pH 7 [22]. At pH 7 a higher loading (*q* = 17.7 mg/g) was achieved from swine liquid manure as compared to the arbitrary pH 8.2 (*q* = 12.5 mg/g) [17]. Furthermore, investigations with artificial swine wastewater stated that most ammonium was removed at pH 7, also showing a strong decrease in sorption with increasing pH [39]. On the contrary, the sorption of ammonium from drinking water was not affected by pH in the range of 5–9 [40].

The results here reveal that at a pH of 7, ammonium is eliminated to a high degree. In contrast to the literature results, a high degree of elimination could be realized with CCP 20 at arbitrary pH (7.9 or 8.0). A higher pH, however, should be avoided.

### 3.2. Isothermal Adsorption

**Figure 3:**
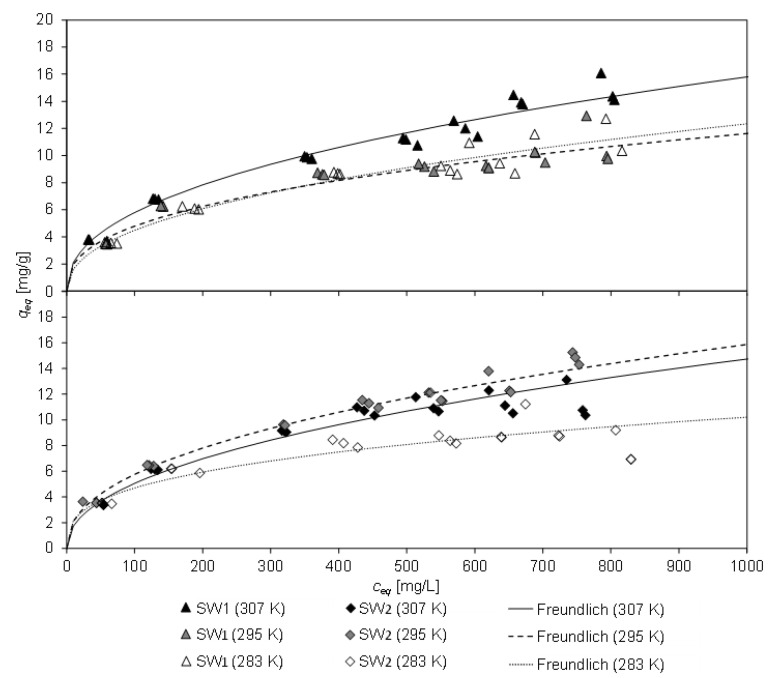
Equilibrium load *q_eq_* and equilibrium concentration *c_eq_* of CCP 20 and Freundlich isotherm of CCP 20 after 20 h contact time with SW1 or SW2 at different temperatures (SW1: *c*_0_ = 967 mg/L NH_4_-N; initial pH 8.1; final pH 7.6–8.6. SW2: *c*_0_ = 866–913 mg/L NH_4_-N; initial pH 7.9; final pH 7.2–8.0).

Figure 3 displays the equilibrium loading *q_eq_* of CCP 20 and the corresponding equilibrium concentration *c_eq_* after 20 h contact time with SW1 and SW2 at different temperatures (283 K, 295 K, and 307 K). The lines represent the Freundlich equation, which gained the highest degree of determination of all tested isothermal equations (Freundlich, Langmuir, Temkin). The coefficients of the isothermal equations and their coefficient of determination are listed in Table 5. From the high concordance with the Freundlich isotherm, it can be deduced, that CLI has a heterogeneous surface, resulting in non-ideal sorption. Furthermore, the sorption process from sludge water is non-ideal, e.g., possible multiple occupancy of a sorption site as well as not all sorption sites are occupied.

The pH of the filtrate of SW1 (initial pH 8.1) changed independently to values between 7.6 and 8.6 during contact, and to 7.2 to 8.0 for SW2 (initial pH 7.9), whereby the former occurred with low sorbent masses and the latter was determined in a blind test without sorbent. As a result, the pH value dropped slightly due to the sorption process.

The highest equilibrium load of CCP 20 with ammonium from SW1 was 16.1 mg/g at 307 K. However, this was considerably lower by 13% than observed for matrix-free solution (NH_4_Cl) with a similar concentration of 1000 mg/L NH_4_-N (*q_eq_* = 18.8 mg/g [10]). For SW2, the highest equilibrium load was 15.3 mg/g at 295 K (18% lower than for matrix-free solution). The load was lower at 307 K, but this was probably due to an alteration in the ammonium concentration in SW2 between the tests.

The minor loading of CCP 20 compared to matrix-free solution could be ascribed to the constituents in the sludge water, which interfere with the sorption process and thus lead to a reduction of the adsorption capacity. In particular, the deeper sorption sites in the framework of the CLI are probably more difficult to access. Access pores may be blocked or sorption sites may be occupied by other constituents in the sludge water. Furthermore, blocking of zeolite pores could be caused e.g., by solids. In addition, the viscosity of the sludge water changes depending on the temperature, which could also influence the loading of the CLI.

A decrease in the adsorption capacity of zeolite due to the wastewater matrix has been reported in several publications [16,17]. By using swine manure (*c*_0_ = 7700 mg/L NH_4_^+^) instead of NH_4_Cl solution (*c*_0_ = 7700 mg/L NH_4_^+^), the uptake capacity of the tested CLI decreased from 10 mg/g to 2 mg/g [17]. To a similar extent as was found in this paper, a decrease of 10–20% of the adsorption capacity when using leachate from a sewage sludge landfill (*c*_0_ = 115.16 mg/L NH_4_-N) instead of NH_4_Cl solution (*c*_0_ = 119.48 mg/L NH_4_-N) was reported [16]. The authors attributed this to ions such as Na^+^, K^+^, Mg^2+^, and Ca^2+^ with ion ratios of ammonium to potassium of [NH_4_^+^]/[K^+^] ≈ 14.6 and sodium of [NH_4_^+^]/[Na^+^] ≈ 2.7 in the leachate. However, in both sludge waters investigated ammonium was present in multiple excess (SW1: [NH_4_^+^]/[K^+^] ≈ 12; [NH_4_^+^]/[Na^+^] ≈ 13; SW2: [NH_4_^+^]/[K^+^] ≈ 24–30; [NH_4_^+^]/[Na^+^] ≈ 10–12) and thus ammonium dominated the sorption process.

The determined ion ratio of ammonium to potassium and sodium in SW1 and SW2 was higher than reported by Wang et al. [16], but the difference in load between sludge water and matrix-free solution [10] was within the same magnitude. Obviously, the capacity of CCP 20 is influenced by both, the cations contained in the sludge water and by the matrix of the sludge water.

### 3.3. Thermodynamic Properties

Table 6 lists the determined thermodynamic state variables free reactivity enthalpy *ΔG*^0^, free standard enthalpy *ΔH^0^,* and molar standard entropy *ΔS*^0^ after 20 h contact time of CCP 20 with SW1 and SW2.

In the examined temperature range (283–307 K), an exergonic, i.e., voluntary sorption process of ammonium to CLI, can be deduced from to the negative free reaction enthalpy *ΔG*^0^. The free standard enthalpy *ΔH*^0^ was positive for SW1, i.e., an endothermic reaction was present. In contrast to this, an exothermic reaction was observed for SW2. The positive molar standard entropy *ΔS*^0^ indicates a directed process. Independent of the matrix, the reaction of ammonium with CLI is voluntary and directed.

From experiments with CLI and matrix-free solution, an exergonic reaction was also reported with *ΔG*^0^ ranging from −2.8662 to 0.22 kJ/mol [41], −0.79 to 1.63 kJ/mol [42], and −0.22 to 1.60 kJ/mol [43]. In this study, the values of *ΔG*^0^ ranged from −15 to −17 kJ/mol. The much lower values regarding *ΔG*^0^ of this study can be attributed to the smaller particle size and therefore short diffusion pathways of cations into the CLI. For their experiments, Alshameri et al. [41], Gunay [42], and Karadag et al. [43] used zeolites with larger particle sizes such as 0.063–0.074 mm, 0.3–0.6 mm, and 1.0–1.4 mm.

On the contrary to the results published by other researchers (*ΔH*^0^: −49.384, −22.34, −5.43, −15.38 kJ/mol [41,42,43,44]), which indicate that adsorption of ammonium is exothermic, a slightly endothermic adsorption from SW1 (*ΔH*^0^: 8.5 kJ/mol (SW1)) was found. However, adsorption from SW2 was exothermic (*ΔH*^0^: −15.5 kJ/mol).

Furthermore, results reported with negative values of *ΔS*^0^ (−0.1561, −43.03, −49.34, −74.42 kJ/(mol K) [41,42,43,44]) indicate decreasing ammonium uptake due to increasing randomness. In contrast to this, a strongly directed adsorption process, as indicated by positive *ΔS*^0^ values ranging between 2.6 J/(K mol)^−1^ (SW2) and 84.3 J/(K mol)^−1^ (SW1), was achieved.

### 3.4. Kinetic Studies

#### 3.4.1. Influence of Temperature on Kinetics

**Figure 4:**
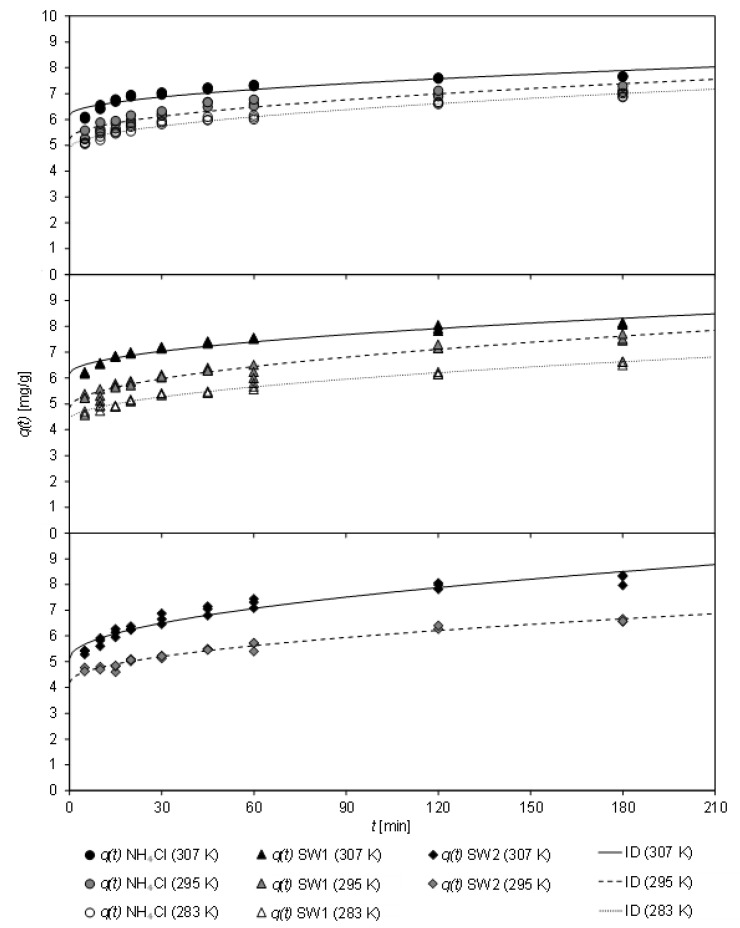
Loading *q*(*t*) of CCP 20 as a function of contact time *t* at different temperatures (283–307 K) and fit to the ID model (specific sorbent ratio: 0.1 g_CLI_/mg_NH4−N_; NH4Cl solution: *c*_0_ = 1000 mg/L NH4-N; initial pH 5.3; final pH 6.3–7.0; SW1: *c*_0_ = 967 mg/L NH4-N; initial pH 7.6; final pH 7.4–8.3; SW2: *c*_0_ = 913 mg/L NH4-N; initial pH 7.6; final pH 6.5–8.2).

In Figure 4, the loading of CCP 20 with ammonium from sludge water at various temperatures (283 K to 307 K) after different contact times (up to 180 min) is depicted. The sorption kinetics at the investigated temperatures are fit to the ID model.

Over the entire temperature range, a rapid adsorption of ammonium by CCP 20 was observed. Within the first five minutes, a high loading occurred, independently of the sludge water matrix.

Table 7 shows the coefficients of the kinetic fit according to both, PSO and ID model, the latter of which achieved a higher coefficient of determination.

From the PSO model, an increase of *k*_2_ from 0.065 g/(mg min) to 0.090 g/(mg min) could be derived with increasing temperature of the matrix-free NH_4_Cl solution. For SW1, *k*_2_ first decreased with increasing temperature from 0.056 g/(mg min) (at 283 K) to 0.043 g/(mg min) (295 K), but then increased to 0.070 g/(mg min) (307 K). With SW2, *k*_2_ remained almost unchanged with 0.041 g/(mg min) (at 295 K) and 0.037 g/(mg min) (at 307 K). Thus, the sludge-water matrix caused a reduction of the sorption rate *k*_2_. Temperature only had a minor effect on the sorption rate in the range of 283–295 K. The equilibrium load *q_e_* was similar for all samples, with a higher value for *q_e_* being obtained with increasing temperature.

For all three matrices, an increase in the initial sorption *C* was observed with increasing temperature, whereas the sorption rate *k_ID_* was only slightly affected. In the case of the matrix-free solution, *k_ID_* decreased from 0.159 mg/(min^0.5^ g) at 283 K to 0.129 mg/(min^0.5^ g) at 307 K. In contrast, *k_ID_* increased slightly with SW1 between 283 K and 295 K. This low coefficient of determination of the sorption kinetics at 283 K indicates that no ID was present at low temperatures. Nevertheless, it was also shown that the kinetics at 295 K slowed down. Here, *k_ID_* decreased from 0.208 mg/(min^0.5^ g) to 0.161 mg/(min^0.5^ g).

Regardless of the sorption kinetics, it should be recognized that ammonium uptake was mainly affected by initial sorption, which in turn was greater at higher temperatures. After the CCP 20 was in contact with the matrix-free solution or sludge waters for 30 min at 307 K, a load between 6.88 mg/g (SW2) and 7.15 mg/g (SW1) was determined.

Similar conclusions regarding a decrease of the sorption rate as a result of an increase in temperature were described according to the PSO model (NH_4_Cl solution) [43]. Furthermore, it was concluded that this was an exothermic process, which was slower due to increased temperatures. On the other hand, it was found that *k*_2_ was reduced and *k_ID_* increased due to increased temperature [23]. Consequently, the equilibrium was reached later. However, the values reported by Erdoğan and Ülkü [23] for *k_ID_* with 4.8 10^−3^ mg/(min^0.5^ g) at 298 K and 5.4 10^−3^ mg/(min^0.5^ g) at 313 K were about a factor of 30 lower than the values obtained with CCP 20. This is probably ascribed to the larger particle sizes of the CLI (0.85–2.00 mm [23]) investigated.

#### 3.4.2. Influence of the Specific Dosage on Kinetics

**Figure 5:**
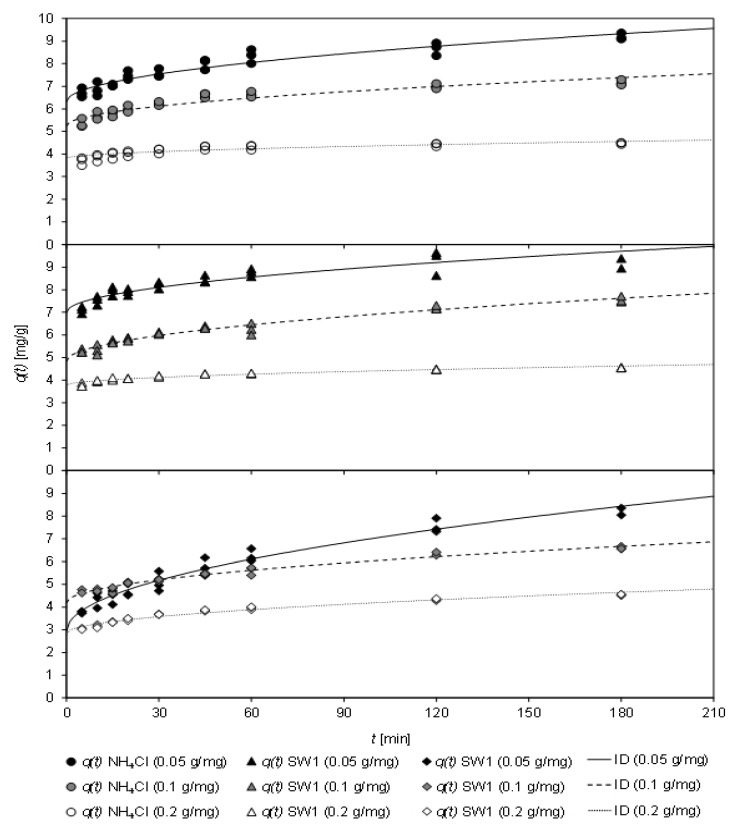
Loading *q*(*t*) of CCP 20 as a function of the contact time *t* after different specific sorbent ratios (0.05–0.2 g_CLI_/mg_NH4-N_) aligned with the ID model (*T* = 295 K; NH4Cl solution: *c*_0_ = 1000 mg/L NH4-N; initial pH 5.3; final pH 6.3–7.0; SW1: *c*_0_ = 967 mg/L NH4-N; initial pH 7.6; final pH 7.5–8.5; SW2: *c*_0_ = 718–913 mg/L NH4-N; initial pH 7.6; final pH 7.7–8.2).

Figure 5 displays the loading *q*(*t*) as a function of the contact time *t* of CCP 20 at different specific sorbent dosages (0.05–0.2 g_CLI_/mg_NH4N_) after contact with NH_4_Cl solution as well as SW1 and SW2. In addition, the fit of the ID model, which had gained the highest coefficient of determination, is shown. A load between 3.07 mg/g and 7.25 mg/g was realized after only 5 min, regardless of the matrix. After 30 min contact time, depending on the specific sorbent addition (0.05–0.2 g_CLI_/mg_NH4N_), a loading of between 4.15 mg/g and 7.56 mg/g was obtained with the NH_4_Cl solution. However, after 60 min, the load increased to values between 4.30 mg/g and 8.34 mg/g and after 120 min to between 4.41 mg/g and 8.66 mg/g.

Table 8 displays the coefficients of the sorption kinetics according to the PSO model and the ID model. The high coefficients of determination of the ID model indicate the limitation of the sorption rate by intraparticle diffusion.

The coefficients of the PSO model and the ID model indicate that the sorbent dosage has a decisive influence on the rate of ammonium uptake. In the investigated samples, *k*_2_ increased with increasing sorbent dosage, i.e., the sorption equilibrium was achieved earlier. With the lowest specific sorbent dosage of 0.05 g_CLI_/mg_NH4-N_, *k*_2_ was between 0.008 g/(mg min) (SW2) and 0.048 g/(mg min) (SW1) and rose disproportionately with a specific dosage of 0.2 g_CLI_/mg_NH4-N_ to 0.062 g/(mg min) (SW2), 0.154 g/(mg min) (NH_4_Cl) or even up to 0.188 g/(mg min) (SW1). In contrast, the load *q_e_* decreased from 9.26 mg/g (SW2) to 4.35 mg/g (SW1) with increasing sorbent dosage. The values for *k_ID_* became smaller with increasing sorbent loading, i.e., the sorption equilibrium was achieved earlier. Thus, *k_ID_* values of 0.410 mg/(min^0.5^ g) (SW2), 0.202 mg/(min^0.5^ g) (SW1), and 0.225 mg/(min^0.5^ g) (NH_4_Cl) where reached at a dosage of 0.05 g_CLI_/mg_NH4-N_, which then decreased to 0.135 mg/(min^0.5^ g) (SW2) and 0.063 mg/(min^0.5^ g) (SW1) and 0.058 mg/(min^0.5^ g) (NH_4_Cl), respectively, at a dosage of 0.2 g_CLI_/mg_NH4-N_. The constant *C*, which is proportional to the thickness of the boundary layer and represents the initial sorption, was also reduced from 6.31 mg/g to 3.78 mg/g (NH_4_Cl), from 7.00 mg/g to 3.77 mg/g (SW1), and from 4.19 mg/g to 2.84 mg/g (SW2) when a larger sorbent dosage (0.05–0.2 g_CLI_/mg_NH4-N_) was applied. This can be ascribed to the fact that with higher specific dosage, more sorption sites are provided, resulting in a rapid sorbent equilibrium but lower load.

During the contact time investigated, only a slight influence of the sample matrix on the load has been observed. Neither was the uptake rate *k*_2_ or *k_ID_* influenced (except for SW2 at a specific dosage of 0.05 g_CLI_/mg_NH4-N_). However, regardless of the matrix, CCP 20 was in equilibrium after 120 min. In contrast, it was reported that for ammonium from matrix-free sorption solution one hour of contact time, but for that from the leachate 2.5 h were needed to achieve equilibrium [16]. The authors ascribed this to interfering cations such as Na^+^, K^+^, Mg^2+^, and Ca^2+^ ([NH_4_^+^]/[Na^+^] ≈ 2.7; [NH_4_^+^]/[K^+^] ≈ 14.6) in the leachate investigated. However, the high stoichiometric excess of ammonium in the sludge waters investigated in this work was larger (SW1: [NH_4_^+^]/[K^+^] ≈ 12; [NH_4_^+^]/[Na^+^] ≈ 13; SW2: [NH_4_^+^]/[K^+^] ≈ 24–30; [NH_4_^+^]/[Na^+^] ≈ 10–12). Hence, the interfering of competing cations can be assumed as low. Therefore, no significant difference between matrix-free solution and sludge water could be determined in this experiment.

#### 3.4.3. Influence of the Pre-load on Sorption Kinetics

During one sorption process, the CCP 20 may not be completely loaded, e.g., if the lowest possible residual concentration is to be achieved by a higher dosage of sorbent. In a process cascade, this partially pre-loaded sorbent could be returned and recontacted with sludge water. Similarly, a partial regeneration of the sorbent can result in partially loaded sorbent.

**Figure 6:**
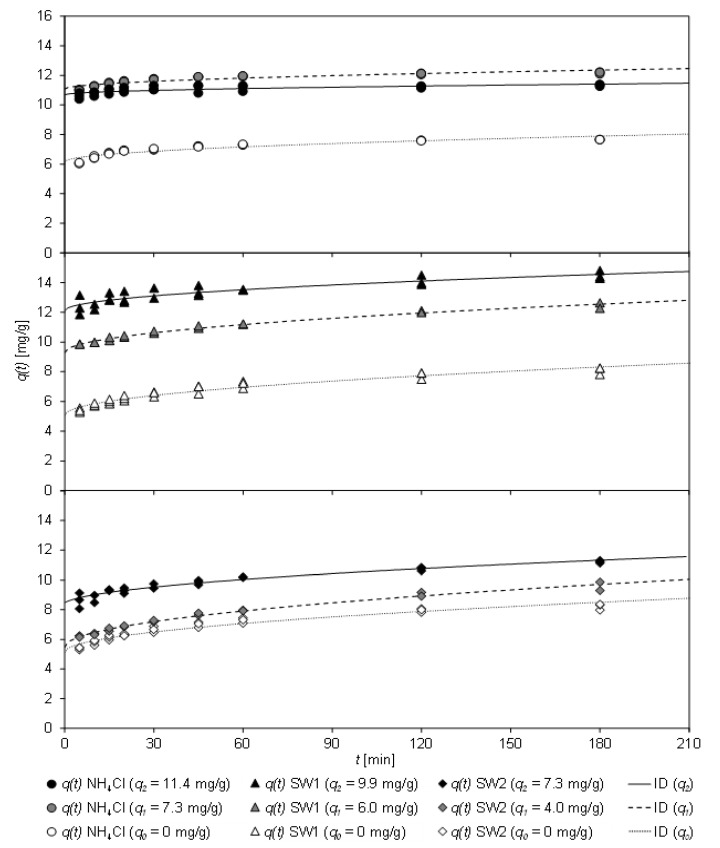
Loading *q*(*t*) of CCP 20 as a function of the contact time *t* of differently pre-loaded CCP 20 (*q_0_–q*_2_ = 0–11.4 mg/g) and fit with the ID model (specific sorbent dosage: 0.1 g_CLI_/mg_NH4-N_; *T* = 307 K; NH4Cl solution: *c*_0_ = 1000 mg/L NH4-N; initial pH 5.3; final pH 6.0; SW1: *c*_0_ = 967 mg/L NH4-N; initial pH 7.9; final pH 7.6; SW2: *c*_0_ = 775–913 mg/L NH4-N; initial pH 7.6; final pH 6.5–8.4).

Figure 6 shows the loading *q*(*t*) of partially loaded CCP 20 after contact with NH_4_Cl solution, SW1, and SW2 as a function of the contact time *t*. For pre-loading, the sorbent was brought into contact with the sample for 30 min (*q*_1_) or 60 min (*q*_2_) at 307 K (based on the results from Section 3.4.1). In addition, the fit of the ID model is shown, which achieved the highest coefficient of determination (Table 9).

As Figure 6 shows, the loading of CCP 20 increased with increasing contact time. Unloaded sorbent is marked as *q*_0_, pre-loaded sorbent as *q*_1_ and *q*_2_, respectively. Pre-loaded CCP 20 (*q*_1_ and *q*_2_) adsorbed additional ammonium on contact with the sample. A considerable change in the load *q*(*t*) occurred for the matrix-free NH_4_Cl solution. However, CCP 20 pre-loaded with 11.4 mg/g was already close to the sorption equilibrium, so that no significant increase in loading could be determined.

In contrast, with both wastewater matrices of the sludge waters SW1 and SW2 even at the highest pre-load (*q_2_(SW1)* = 9.9 mg/g and *q_2_(SW2)* = 7.3 mg/g), an increase of loading was achieved.

Table 9 shows the coefficients of the kinetic fit according to the PSO and ID models, the latter achieving higher coefficients of determination.

Within 5 min contact time, a high initial loading (*C* = 5.12–6.16 mg/g) of the unloaded sorbent (*q*_0_) was achieved, independent of the sample matrix. When partially pre-loaded (*q*_1_), the loading of the sorbent was considerably increased in all tests; depending on the pre-loading (approx. 80% for NH_4_Cl, almost 100% for SW1 and approx. 60% for SW2). In the first pre-loading step (*q*_1_), the initial loading *C* of NH_4_Cl increased significantly from 6.16 mg/g (*q*_0_) by about 80% to 11.07 mg/g (*q*_1_). For SW1, it also increased considerably from 6.16 mg/g (*q*_0_) to 9.30 mg/g (*q*_1_), but for SW2 only slightly from 5.12 mg/g (*q*_0_) to 5.46 mg/g (*q*_1_). In case of the highest preload (*q*_2_), the initial load *C* of matrix-free NH_4_Cl was lower (10.69 mg/g), but in case of sludge water it increased (SW1: 12.11 mg/g; SW2: 8.25 mg/g). The decreasing values for *k_ID_* of SW1 (*q*_1_–*q*_2_: 0.243–0.182 mg/(min^0.5^ g)) and SW2 (*q*_1_–*q*_2_: 0.316–0.229 mg/(min^0.5^ g)) indicate that the sorbent reached equilibrium faster due to the partial pre-loading. After 30 min, preloaded (*q*_2_) CCP 20 was loaded to an extend between 9.45 mg/g (SW2) and 13.63 mg/g (SW1).

The fit of the sorption kinetics from matrix-free NH_4_Cl solution by means of the PSO model reveals that *k*_2_ increased with increasing pre-load of the sorbent, i.e., the sorbent achieved equilibrium faster. However, *k*_2_ of unloaded CCP 20 (*q*_0_) attained 0.090 g/(mg min), which increased to 0.135 g/(mg min) when partially loaded (*q*_1_) and finally to 0.161 g/(mg min) with the highest pre-load (*q*_2_), which was almost in equilibrium. On the contrary, the *k*_2_ values for SW1 (*q*_0_–*q*_2_: 0.042–0.070 g/(mg min)) and SW2 (*q*_0_–*q*_2_: 0.027–0.043 g/(mg min)) do not allow clear conclusions due to their wide variation.

Nevertheless, it has been ascertained that the sorption equilibrium is achieved faster from matrix-free NH_4_Cl solution than from sludge water. This can be ascribed to cations competing for sorption sites, but also to organic matter or particles contained in the sludge water and the slower diffusion of ammonium to deeper sorption sites. In a process cascade, in which a high CLI dosage has to achieve the lowest possible residual concentration, the partially loaded CLI could be brought into contact with sludge water again in order to use its capacity to the full extent. In order to achieve the highest possible loading of the sorbent, up to three sorption phases should be carried out, each lasting a maximum of 30 min.

## 4. Conclusions

From experiments with high strength sludge waters with ammonium concentrations from 718 mg/L NH_4_-N to 967 mg/L NH_4_-N by means of Carpathian clinoptilolite, the following boundary conditions can be derived with which the highest possible loading of the sorbent CCP 20 can be achieved:

- the pH value should be in a range of 2 to 8 (or the arbitrary pH if below 8),
- the temperature of 307 K should be preferred over lower temperatures (e.g., 283 K or 295 K),
- the choice of a low specific sorbent dose (e.g., <0.1 g_CLI_/mg_NH4-N_) is advantageous,
- pre-loaded sorbent should be recontacted with the sludge water several times (up to three times)
- the contact time of (pre-loaded) sorbent should be at least 30 min.

However, other boundary conditions may be relevant, depending on the objectives of the treatment, e.g., high loading of the sorbent, shortest possible contact time, low effluent concentrations. For the design as well as the implementation of the process, the required contact time is of major importance. In the experiments it could be shown that a high loading of the clinoptilolite can be achieved already after 30 min. Therefore, it can be deduced that the necessary equipment for the treatment of the relatively small partial flow of the sludge water compared to the main wastewater flow would only require minor construction and plant engineering upgrades.

Based on the found conditions, it is of interest for future investigations under which parameters the clinoptilolite can be regenerated and possibly reused. Furthermore, it should not be omitted that the liquid resulting from the regeneration is still usable or the recovered ammonium is available in a usable form.

## Figures and Tables

**Table 1:** Physical properties and constituents of the examined sludge waters.

Parameter	Unit	SW1	SW2	NH_4_Cl
pH	–	7.9	8.0	5.3
Conductivity (298 K)	mS/cm	7.10	7.98	9.60
SS	mg/L	195	71–212	–
COD	mg/L	517	340–362	–
COD dissolved	mg/L	304	279–300	–
NH_4_-N	mg/L	967	718–927	1000
NO_3_-N	mg/L	<2	<1	–
NO_2_-N	mg/L	<0.10	3.49	–
PO_4_-P	mg/L	45.7	0.75–4.4	–
F^−^	mg/L	n.d.	1.49	–
Cl^−^	mg/L	174	266–292	–
Br^−^	mg/L	<0.30	0.93	–
SO_4_^2−^	mg/L	~30	< 100	–

n.d.: not detected.

**Table 2:** Elementary composition of the examined slugde waters.

Element	Unit	SW1	SW2
B	mg/L	0.5	0.294–0.390
Na	mg/L	126	129–132
Mg	mg/L	49.5	52.0–62.4
Al	mg/L	0.472/0.823	0.163–1.05
K	mg/L	234	87.5–91.0
Ca	mg/L	65	137–146
Cr	mg/L	0.002	<0.0001
Fe	mg/L	7.6	13.6
Ni	mg/L	0.15	0.030–0.042
Cu	mg/L	0.015	0.012–0.026
Zn	mg/L	n.d.	0.036–0.060
As	mg/L	<0.2	<0.0001
Se	mg/L	0.12	n.d.
Rb	mg/L	0.163	0.070
Sr	mg/L	0.075	0.721–0.770
Cd	mg/L	<0.02	<0.0001
Cs	mg/L	0.024	<0.003
Ba	mg/L	<0.02	0.012
Hg	mg/L	<0.02	<0.0001
Tl	mg/L	<0.02	<0.0001
Pb	mg/L	<0.005	0.002

n.d.: not detected.

**Table 3:** Linear forms of the Langmuir isotherm (according to [28]).

Type	Linear Form	Plot	K_L_	q_max_
I	$\frac{c_{eq}}{q_{eq}}=\frac{1}{q_{max}K_{L}}+\frac{c_{eq}}{q_{max}}$	$\frac{c_{eq}}{q_{eq}}\;\mathrm{vs}.\;c_{eq}$	slope/intercept	1/slope
II	$\frac{1}{q_{eq}}=\left[\frac{1}{q_{max}K_{L}}\right]\frac{1}{c_{eq}}+\frac{1}{q_{max}}$	$\frac{1}{q_{eq}}\mathrm{vs}.\;\frac{1}{c_{eq}}$	intercept/slope	1/intercept
III	$q_{eq}=q_{max}-\left[\frac{1}{K_{L}}\right]\frac{q_{eq}}{c_{eq}}$	$q_{eq}\;\mathrm{vs}.\;\frac{q_{eq}}{c_{eq}}$	1/slope	intercept
IV	$\frac{q_{eq}}{c_{eq}}=q_{max}K_{L}-c_{eq}K_{L}$	$\frac{q_{eq}}{c_{eq}}\;\mathrm{vs}.\;q_{eq}$	(−1) slope	intercept/slope

**Table 4:** Linear forms of PSO model (according to [37]).

Type	Linear Form	Plot	*k*_2_	*q_e_*
I	$\;\frac{t}{q(t)}=\frac{1}{k_{2}q_{e}^{2}}+\frac{t}{q_{e}}$	$\frac{t}{q(t)}\;\mathrm{vs}\;t$	(slope)^2^/intercept	1/slope
II	$\frac{1}{q(t)}=\left[\frac{1}{k_{2}q_{e}^{2}}\right]\frac{1}{t}+\frac{1}{q_{e}}$	$\frac{1}{q(t)}\;\mathrm{vs}\;\frac{1}{t}$	(intercept)^2^/slope	1/intercept
III	${\;q(t)=q}_{e}-\left[\frac{1}{k_{2}q_{e}}\right]\frac{q(t)}{t}$	$q(t)\;\mathrm{vs}\frac{q(t)}{t}$	(−1)/(slope × intercept)	intercept
IV	$\frac{q(t)}{t}{=k}_{2}q_{e}^{2}-\;kq_{e}q(t)$	$\frac{q(t)}{t}\;\mathrm{vs}\;q(t)$	(slope)^2^/intercept	−intercept/slope

**Table 5:** Coefficients of isothermal adaptation according to Freundlich, Langmuir and Temkin for CCP 20 after 20 h contact time with SW1 or SW2 at different temperatures (SW1: *c*_0_ = 967 mg/L NH_4_-N; initial pH 8.1; final pH 7.6–8.6. SW2: *c*_0_ = 866–913 mg/L NH_4_-N; initial pH 7.9; final pH 7.2–8.0).

	Temperature	Freundlich			Langmuir			Temkin		
	*T*	*K_F_*	*1/n*	*r²*	*K_L_*	*q_max_*	*r²*	*A_T_*	*b_T_*	*r²*
	[K]	[L/g]	[−]	[−]	[L/mg]	[mg/g^−^]	[−]	[L/mg]	[J g /(mg mol)]	[−]
SW1	283	0.590	0.440	0.9087	0.005	12.38	0.5351	0.0495	846	0.8989
	295	0.811	0.385	0.9006	0.007	11.42	0.5202	0.0716	954	0.9180
	307	0.777	0.436	0.9603	0.005	17.31	0.5377	0.0631	735	0.9173
SW2	283	0.998	0.337	0.6701	0.006	11.68	0.5137	0.1188	1209	0.6828
	295	0.759	0.440	0.9711	0.005	16.64	0.5386	0.0670	724	0.9311
	307	0.596	0.464	0.8964	0.005	15.08	0.5257	0.0549	798	0.9322

**Table 6:** Thermodynamic properties of CCP 20 after 20 h contact time with SW1 or SW2 (SW1: *c*_0_ = 967 mg L^−1^ NH_4_-N; initial pH 8.1; final pH 7.6–8.6. SW2: *c*_0_ = 866–913 mg L^−1^ NH_4_-N; initial pH 7.9; final pH 7.2–8.0).

Sorptive	Temperature	Free Reaction Enthalpy	Free Standard Enthalpy	Molar Standard Entropy
	*T*	*ΔG* ^0^	*ΔH* ^0^	*ΔS* ^0^
[−]	[K]	[kJ/mol]	[kJ/mol]	[J/(K mol)]
	283	−15.0		
SW1	295	−16.4	8.5	84.3
	307	−17.0		
	283	−16.2		
SW2	295	−16.3	−15.5	2.6
	307	−16.3		

**Table 7:** Coefficients of the sorption kinetics according to the PSO and ID models of CCP 20 at different temperatures (283–307 K, specific sorbent ratio: 0.1 g_CLI_/mg_NH4−N_; NH4Cl solution: *c*_0_ = 1000 mg/L NH4-N; initial pH 5.3; final pH 6.3–7.0; SW1: *c*_0_ = 967 mg/L NH4-N; initial pH 7.6; final pH 7.4–8.3; SW2: *c*_0_ = 913 mg/L NH4-N; initial pH 7.6; final pH 6.5–8.2).

Temperature	Sorptive	Pseudo-Second-Order			Intraparticle Diffusion		
***T***		*k*_2_	*q_e_*	*r* ^2^	*k_ID_*	*C*	*r* ^2^
[K]	[−]	[g/(mg min)]	[mg/g]	[−]	[mg/(min^0.5^ g)]	[mg/g]	[−]
283		0.065	6.60	0.6853	0.159	4.88	0.9642
295	NH_4_Cl	0.064	6.99	0.8319	0.159	5.25	0.8852
307		0.090	7.53	0.9153	0.129	6.16	0.8467
283		0.056	6.23	0.6116	0.172	4.32	0.5236
295	SW_1_	0.043	7.19	0.7486	0.208	4.84	0.9566
307		0.070	7.87	0.8641	0.161	6.16	0.9228
295	SW_2_	0.041	6.34	0.7231	0.185	4.19	0.9704
307		0.037	7.89	0.8370	0.252	5.12	0.9386

**Table 8:** Coefficients of the sorption kinetics according to the PSO and ID models of CCP 20 after different sorbent loads (*T* = 295 K; NH4Cl solution: *c*_0_ = 1000 mg/L NH4-N; initial pH 5.3; final pH 6.3–7.0; SW1: *c*_0_ = 967 mg/L NH4-N; initial pH 7.6; final pH 7.5–8.5; SW2: *c*_0_ = 718–913 mg/L NH4-N; initial pH 7.6; final pH 7.7–8.2.

Specific Sorbent Dosage	Sample	Pseudo-Second-Order			Intraparticle Diffusion		
*m_S_*		*k*_2_	*q_e_*	*r²*	*k_ID_*	*C*	*r²*
[g_CLI_/mg_NH4N_]		[g/(mg min)]	[mg/g]	[−]	[mg/(min^0.5^ g)]	[mg/g]	[−]
0.05		0.041	8.84	0.7704	0.225	6.31	0.9119
0.1	NH_4_Cl	0.064	6.99	0.8319	0.159	5.25	0.8852
0.2		0.154	4.45	0.7605	0.058	3.78	0.7595
0.05		0.048	9.26	0.7849	0.202	7.00	0.8543
0.1	SW1	0.043	7.19	0.7486	0.208	4.84	0.9566
0.2		0.188	4.45	0.8355	0.063	3.77	0.9134
0.05		0.008	8.56	0.8305	0.410	2.93	0.9500
0.1	SW2	0.020	6.78	0.7309	0.185	4.19	0.9704
0.2		0.062	4.35	0.8263	0.135	2.84	0.9647

**Table 9:** Coefficients of the sorption kinetics according to the PSO and ID models of CCP 20 with different pre-loads (specific sorbent dosage: 0.1 g_CLI_/mg_NH4-N_; *T* = 307 K; NH4Cl solution: *c*_0_ = 1000 mg/L NH4-N; initial pH 5.3; final pH 6.0; SW1: *c*_0_ = 967 mg/L NH4-N; initial pH 7.9; final pH 7.6; SW2: *c*_0_ = 775–913 mg/L NH4-N; initial pH 7.6; final pH 6.5–8.4).

Pre-Load		Matrix	Pseudo-Second-Order			Intraparticle Diffusion		
			*k*_2_	*q_e_*	*r²*	*k_ID_*	*C*	*r²*
[mg/g^−1^]		[−]	[g/(mg min)]	[mg/g]	[−]	[mg/(min^0.5^ g)]	[mg/g]	[−]
*q*_0_	0		0.090	7.53	0.9153	0.129	6.16	0.8467
*q*_1_	7.3	NH_4_Cl	0.135	12.08	0.8943	0.096	11.07	0.8302
*q*_2_	11.4		0.161	11.37	0.7261	0.054	10.69	0.5191
*q*_0_	0		0.070	7.87	0.8641	0.161	6.16	0.9228
*q*_1_	6.0	SW1	0.042	12.00	0.7325	0.243	9.30	0.9853
*q*_2_	9.9		0.050	14.32	0.6985	0.182	12.11	0.7495
*q*_0_	0		0.037	7.89	0.8370	0.252	5.12	0.9386
*q*_1_	4.0	SW2	0.027	9.06	0.7603	0.316	5.46	0.9846
*q*_2_	7.3		0.043	10.85	0.5479	0.229	8.25	0.5210

**Figure A1:**
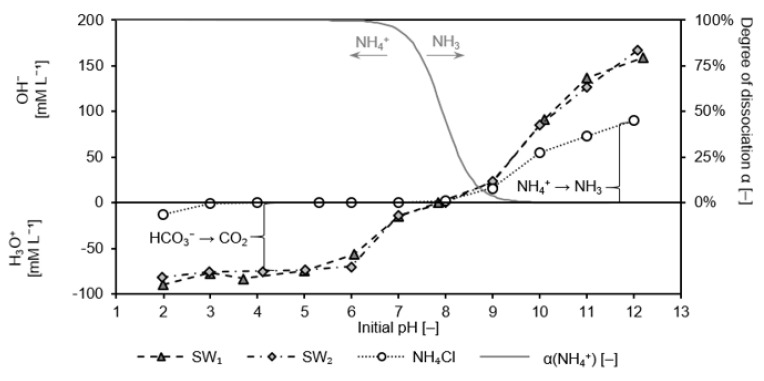
Quantity of OH^−^ (NaOH) and H_3_O^+^ (HCl) to adjust the pH of SW1, SW2, and NH_4_Cl solution as well as the degree of dissociation of ammonium depending on the pH.

## Data Availability

All data comes from the authors.

## Appendix A. Titration Curve of SW1, SW2, and NH_4_Cl Solution

During anaerobic digestion, large quantities of methane and CO_2_ are formed, resulting in high concentrations of hydrogen carbonate in the sludge water. By lowering the pH, the hydrogen carbonate is converted into CO_2_, which then outgasses.

The base capacity, i.e., the amount of OH^−^ required to adjust the pH to 8.2, corresponds to the amount of free CO_2_ in the solution. The acid capacity, i.e., the amount of H_3_O^+^ necessary to adjust the pH to 4.3, corresponds to the amount of HCO_3_^−^. In addition, other compounds such as ammonium, borate, phosphate, sulfate, nitrate, etc. may react, therefore the titration method can only be applied to sludge water to a limited extent [45].

The amounts of OH^−^or H_3_O^+^ required to adjust the pH of SW1, SW2 and NH_4_Cl solution are shown in Figure A1.

Only a small amount of H_3_O^+^ (pH 4: 0.09 mmol/L; pH 3: 1.2 mmol/L; pH 2: 12.7 mmol/L) was required to lower the pH of NH_4_Cl (arbitrary pH 5.3), as no hydrogen carbonate was present. For an increase of the pH, a larger amount of OH^−^ was required due to the conversion of NH_4_^+^ into NH_3_ (pH 7: 0.3 mmol/L; pH 8: 15.8 mmol/L; pH 12: 90 mmol/L). Since no other substances were present in the matrix-free solution, a stoichiometric transformation of OH^−^ for the conversion of NH_4_^+^ into NH_3_ can be assumed when adjusting the pH value.

For both sludge waters SW1 and SW2, almost the same amount of H_3_O^+^ and OH^−^ was required. However, the amount of H_3_O^+^ and OH^−^ required for pH adjustment of NH_4_Cl solution was much lower. Obviously, a large amount of hydrogen carbonate buffering the pH was present. For example, 74.5 mmol/L of H_3_O^+^ was needed to set up a pH of 5 for SW1 and 73.8 mmol/L of H_3_O^+^ for SW2. To adjust a pH of 10, 90.9 mmol/L and 85 mmol/L OH^−^, respectively, were required. Due to the high concentration of interfering ions, it is not possible to determine the exact acid/base capacity and hydrogen carbonate concentration in the sludge waters. Based on the data, it can be estimated at approx. 75–80 mmol/L. The hydrogen carbonate is opposed by a sufficiently large amount of ammonium cations (55–69 mmol/L) as counter ion.

## References

[1] United Nations (2017). World Population Prospects: The 2017 Revision, Key Findings and Advance Tables.

[2] FAO (2017). The Future of food and Agriculture—Trends and Challenges.

[3] Erisman J.W., Sutton M.A., Galloway J., Klimont Z., Winiwarter W. (2008). How a century of ammonia synthesis changed the world. Nat. Geosci..

[4] Appl M. (2000). Ammonia. Ullmann’s Encyclopedia of Industrial Chemistry: Ammonia.

[5] Dawson C.J., Hilton J. (2011). Fertiliser availability in a resource-limited world: Production and recycling of nitrogen and phosphorus. Food Policy.

[6] Janus H.M., van der Roest H.F. (1997). Don’t reject the idea of treating reject water. Water Sci. Technol..

[7] Sengupta S., Nawaz T., Beaudry J. (2015). Nitrogen and Phosphorus Recovery from Wastewater. Curr. Pollut. Rep..

[8] Englert A.H., Rubio J. (2005). Characterization and environmental application of a Chilean natural zeolite. Int. J. Min. Process.

[9] Weatherley L.R., Miladinovic N.D. (2004). Comparison of the ion exchange uptake of ammonium ion onto New Zealand clinoptilolite and mordenite. Water Res..

[10] Wasielewski S., Rott E., Minke R., Steinmetz H. (2018). Evaluation of Different Clinoptilolite Zeolites as Adsorbent for Ammonium Removal from Highly Concentrated Synthetic Wastewater. Water.

[11] Cyrus J.S., Reddy G.B. (2011). Sorption and desorption of ammonium by zeolite: Batch and column studies. J. Env. Sci. Health.

[12] Guaya D., Valderrama C., Farran A., Armijos C., Cortina J.L. (2015). Simultaneous phosphate and ammonium removal from aqueous solution by a hydrated aluminum oxide modified natural zeolite. Chem. Eng. J..

[13] Karadag D., Tok S., Akgul E., Turan M., Ozturk M., Demir A. (2008). Ammonium removal from sanitary landfill leachate using natural Gördes clinoptilolite. J. Hazard. Mater..

[14] Zhang H., Li A., Zhang W., Shuang C. (2016). Combination of Na-modified zeolite and anion exchange resin for advanced treatment of a high ammonia-nitrogen content municipal effluent. J. Colloid Interface Sci..

[15] Du Q., Liu S., Cao Z., Wang Y. (2005). Ammonia removal from aqueous solution using natural Chinese clinoptilolite. Sep. Purif. Technol..

[16] Wang Y., Liu S., Xu Z., Han T., Chuan S., Zhu T. (2006). Ammonia removal from leachate solution using natural Chinese clinoptilolite. J. Hazard. Mater..

[17] Montegut G., Michelin L., Brendle J., Lebeau B., Patarin J. (2016). Ammonium and potassium removal from swine liquid manure using clinoptilolite, chabazite and faujasite zeolites. J. Env. Manag..

[18] Martins T.H., Souza T.S.O., Foresti E. (2017). Ammonium removal from landfill leachate by Clinoptilolite adsorption followed by bioregeneration. J. Env. Chem. Eng..

[19] Farkas A., Rozic M., Barbaric-Mikocevic Z. (2005). Ammonium exchange in leakage waters of waste dumps using natural zeolite from the Krapina region, Croatia. J. Hazard. Mater..

[20] Ji Z.Y., Yuan J.S., Li X.G. (2007). Removal of ammonium from wastewater using calcium form clinoptilolite. J. Hazard. Mater..

[21] Lin L., Lei Z., Wang L., Liu X., Zhang Y., Wan C., Lee D., Tay J.H. (2013). Adsorption mechanisms of high-levels of ammonium onto natural and NaCl-modified zeolites. Sep. Purif. Technol..

[22] Temel F.A., Kuleyin A. (2016). Ammonium removal from landfill leachate using natural zeolite: Kinetic, equilibrium, and thermodynamic studies. Desalination Water. Treat..

[23] Erdoğan B.C., Ülkü S. (2011). Ammonium sorption by Gördes clinoptilolite rich mineral specimen. Appl. Clay Sci..

[24] Freundlich H. (1906). Over the adsorption in solution. J. Phys. Chem..

[25] Ho Y.-S., Porter J.F., McKay G. (2002). Equilibrium isotherm studies for the sorption of divalent metal ions onto peat: Copper, nickel and lead single component systems. Water Air Soil Pollut..

[26] Langmuir I. (1918). The Adsorption of Gases on Plane Surfaces of Glass, Mica and Platinum. J. Am. Chem. Soc..

[27] Kinniburgh D.G. (1986). General purpose adsorption isotherms. Env. Sci. Technol..

[28] Chen X. (2015). Modeling of Experimental Adsorption Isotherm Data. Information.

[29] Temkin M.I., Pyzhev V. (1940). Kinetics of ammonia synthesis on promoted iron catalyst. Acta Physicochim. USSR.

[30] Dada A.O., Olalekan A.P., Olatunya A.M., Dada O. (2012). Langmuir, Freundlich, Temkin and Dubinin–Radushkevich Isotherms Studies of Equilibrium Sorption of Zn^2+^ Unto Phosphoric Acid Modified Rice Husk. IOSR-JAC.

[31] Aharoni C., Ungarish M. (1977). Kinetics of activated chemisorption. Part 2—Theoretical models. J. Chem. Soc. Faraday Trans. 1 Chem. Condens. Phases.

[32] Milonjic S. (2007). A consideration of the correct calculation of thermodynamic parameters of adsorption. J. Serb. Chem. Soc..

[33] Weber W.J., Morris J.C. (1963). Kinetics of Adsorption on Carbon from Solution. J. Sanit. Eng. Div..

[34] Qiu H., Lv L., Pan B., Zhang Q., Zhang W. (2009). Critical review in adsorption kinetic models. J. Zhejiang Univ. Sci. A.

[35] McKay G., Otterburn M.S., Sweeney A.G. (1980). The removal of colour from effluent using various adsorbents, III. Silica: Rate Processes. Water Res..

[36] Ho Y.-S., McKay G. (1998). A Comparison of Chemisorption Kinetic Models Applied to Pollutant Removal on Various Sorbents. Process Saf. Environ. Prot..

[37] Ho Y.-S. (2006). Second-order kinetic model for the sorption of cadmium onto tree fern: A comparison of linear and non-linear methods. Water Res..

[38] (1983). Deutsches Institut für Normung e.V., 38406–5. German Standard Methods for the Examination of Water, Waste Water and Sludge.

[39] (2009). Deutsches Institut für Normung e.V., 10304–1. Water Quality—Determination of Dissolved Anions by Liquid Chromatography of Ions —Part 1: Determination of Bromide, Chloride, Fluoride, Nitrate, Nitrite, Phosphate and Sulfate.

[40] Li M., Zhu X., Zhu F., Ren G., Cao G., Song L. (2011). Application of modified zeolite for ammonium removal from drinking water. Desalination.

[41] Alshameri A., Yan C., Al-Ani Y., Dawood A.S., Ibrahim A., Zhou C., Wang H. (2014). An investigation into the adsorption removal of ammonium by salt activated Chinese (Hulaodu) natural zeolite: Kinetics, isotherms, and thermodynamics. J. Taiwan Inst. Chem. Eng..

[42] Gunay A. (2007). Application of nonlinear regression analysis for ammonium exchange by natural (Bigadic) clinoptilolite. J. Hazard. Mater..

[43] Karadag D., Koc Y., Turan M., Armagan B. (2006). Removal of ammonium ion from aqueous solution using natural Turkish clinoptilolite. J. Hazard. Mater..

[44] Tosun I. (2012). Ammonium removal from aqueous solutions by clinoptilolite: Determination of isotherm and thermodynamic parameters and comparison of kinetics by the double exponential model and conventional kinetic models. Int. J. Env. Res. Public Health.

[45] (2005). Deutsches Institut für Normung e.V., 38409–7. German Standard Methods for the Examination of Water, Waste Water and Sludge—General Measures of Effects and Substances (Group H)—Part 7: Determination of Acid and Base Capacity (H 7).

